# The Effect of Ultrasonic Agitation on the Porosity Distribution in Apically Perforated Root Canals Filled with Different Bioceramic Materials and Techniques: A Micro-CT Assessment

**DOI:** 10.3390/jcm10214977

**Published:** 2021-10-27

**Authors:** Saulius Drukteinis, Goda Bilvinaite, Hagay Shemesh, Paulius Tusas, Vytaute Peciuliene

**Affiliations:** 1 Institute of Dentistry, Faculty of Medicine, Vilnius University, Zalgirio 115, LT-08217 Vilnius, Lithuania; goda.bilvinaite@gmail.com (G.B.); paulius.tusas@gmail.com (P.T.); vytaute.peciuliene@mf.vu.lt (V.P.); 2 Academic Centre for Dentistry Amsterdam (ACTA), Gustav Mahlerlaan 3044, 1081 LA Amsterdam, The Netherlands; h.shemesh@acta.nl

**Keywords:** apical plug, BioRoot RCS, micro-computed tomography, MTA Flow, porosity, root perforation, single cone, ultrasonic

## Abstract

The present study evaluated the effect of ultrasonic agitation on the porosity distribution of BioRoot RCS/single gutta-percha cone (BR/SC) and MTA Flow (MF) root canals fillings used as apical plugs in moderately curved and apically perforated roots. Eighty mesial root canals of mandibular first molars were enlarged up to ProTaper NEXT X5 rotary instrument 2 mm beyond the apical foramen, simulating apical perforations. Specimens were randomly divided into four experimental groups (20 canals per group) according to the material and technique used for root canal obturation: BR/SC, BR/SC with ultrasonic agitation (BR/SC-UA), MF and MF with ultrasonic agitation (MF-UA). The ultrasonic tip was passively inserted into the root canal after the injection of flowable cement and activated for 10 s. The specimens were scanned before and after obturation with a high-resolution micro-computed tomography scanner, and the porosity of the apical plugs was assessed. The differences between groups were analyzed using the Kruskal-Wallis and Mann-Whitney tests, with the significance level set at 5%. None of the obturation materials and techniques used in this study was able to provide a pore-free root canal filling in the apical 5 mm. Considerably higher percentages of open and closed pores were observed in the MF and MF-UA groups, with the highest porosity being in the MF-UA group (*p* < 0.05). No significant differences were observed between the BR/SC and BR/SC-UA groups, where the quantity of open and closed pores remained similar (*p* > 0.05).

## 1. Introduction

Root perforation is characterized as a communication between the root canal system and the surrounding periodontal tissues [1]. Perforations occurring due to dental caries or resorption are commonly defined as pathologic in nature [2], while iatrogenic perforations are usually related to inappropriate prosthodontic or endodontic treatment [3]. Up to 20% of endodontically treated teeth are diagnosed with root perforations, of which the majority are caused by various iatrogenic errors [4]. The most severe complication of root perforations is a persistent inflammation, breakdown of periodontal tissues and subsequent loss of bone attachment, ultimately leading to a tooth extraction [5]. Therefore, early diagnosis and appropriate perforation repair have a major influence on the long-term prognosis and survival of the affected tooth [4]. It is generally assumed that apical root perforation, which usually occurs because of endodontic instrumentation during the root canal preparation, has a good prognosis [6]. However, the management of apical root perforations frequently poses a challenge even for experienced endodontists, as visualization and direct access to the perforation site, especially in moderately or severely curved root canals, can be remarkably complicated, with a significant risk of collateral treatment mishaps, errors and complications [2].

The main goal of apical root perforation repair is to obtain a persistent bacteria-tight apical seal to prevent the percolation of fluids, microorganisms and their byproducts in the periapical tissues, allowing the healing and reorganization of damaged tissues [7]. Before the introduction of mineral trioxide aggregate (MTA), various dental materials, such as amalgam or glass ionomer cement, were used to repair root perforations. However, MTA instantly gained popularity due to its favorable biological, physical and chemical properties, which ensured an overall success rate of perforation repair of more than 80% [3,7]. Nevertheless, modifications of the original MTA formulation have been recently made to overcome its poor handling characteristics and long setting time [8]. MTA Flow (MF) (Ultradent Products Inc., South Jordan, UT, USA) is a relatively new MTA-based repair material, consisting of a di- and tri-calcium silicate grey powder and a water-soluble silicone-based gel [9]. MF was developed to give the clinician a variety of mixing options and consistencies, facilitating the manipulation and delivery of the material into the root canal [10]. Due to the extremely small particle size of less than 10μm, MF can be prepared in a thin consistency and delivered to the perforation site using a 29-G needle [11].

Although MTA-based materials have been widely used for root perforation repair since their first introduction [12], various investigations of hydraulic calcium silicate-based cements (HCSC) have shown that BioRoot RCS (Septodont, Saint-Maur-des-Fosses, France) could be effectively used as a filler and seal the apical root perforation as well [13]. BioRoot RCS possesses all the necessary antibacterial, biocompatible and bioactive properties, which promote the regeneration of periapical tissues and contribute to the recruitment of osteo-odontogenic stem cells within the apical environment [14]. Moreover, this material has the desirable dimensional stability and low solubility and provides high clinical success rates when used in conjunction with a single gutta-percha cone (SC) obturation technique [15,16,17]. In contrast to cold lateral compaction or various thermoplastic methods, the BioRoot RCS/single gutta-percha cone (BR/SC) obturation technique is clinically appealing due to its simplicity, as no superior clinical skills or any additional armamentarium and devices are needed [18]. However, the available data on the performance of the BR/SC technique used for an apical plug in apically perforated roots are still limited. There is only one study demonstrating the sealability of apical perforations using the BR/SC technique and porosity distribution in these fillings [19].

Ultrasonic devices have been successfully used in endodontics over the years for a wide range of clinical procedures, including root canal obturation [20,21]. It has been reported that ultrasonication of the sealers during the root canal filling procedure may increase their penetrability into the dentinal tubules and improve the interfacial adaptation between the filling material and the root canal wall [22,23]. Additionally, ultrasonic energy is capable of rearranging the material particles and removing the entrapped air and thus reducing the porosity [24,25]. Therefore, ultrasonic agitation has been recommended in order to improve the quality and homogeneity of root canal fillings [25,26]. However, most of the previous research has investigated the effect of ultrasonic agitation, applied to the sealers indirectly, and there are still no data available on the porosity distribution within the BR/SC and MF root canal fillings after the use of direct ultrasonication.

Micro-computed tomography (micro-CT) is a widely accepted non-destructive method to perform two-dimensional (2D) and three-dimensional (3D) assessments of root canal fillings using high-resolution images [27]. Micro-CT analysis, due to its high accuracy, can be used to determine the overall porosity of the fillings as well as to identify and quantify open and closed pores separately [28]. Therefore, the present study aimed to evaluate, by means of micro-CT analysis, the effect of direct ultrasonic agitation on the porosity distribution in BR/SC and MF root canal fillings used as apical plugs in artificially perforated and moderately curved roots of mandibular molars. The null hypothesis tested was that direct ultrasonic agitation significantly impacts the quality and homogeneity of BR/SC and MF apical plugs, decreasing their porosity.

## 2. Materials and Methods

### 2.1. Specimen Selection and Preparation

A total of 40 human mandibular first molars were selected for this study, under the approval of the local ethics committee (protocol no. EK-2). The minimum sample size was calculated using G*Power 3.1.9.7 software (Heinrich Heine, Iniversität Düsseldorf, Düsseldorf, Germany) followed by t-test family, α error probability of 0.05 and 1-β error probability of 0.95. Therefore, the requirement of 16 root canals per group was determined. Teeth were extracted for medical reasons unrelated to the present study and were stored in a saline solution at room temperature until use. Only molars with two separate mesial root canals, fully developed root apices and moderately curved roots (10°–20°) were selected. The root curvature was determined on preoperative radiographs using Shilder’s method [29].

The orifices of root canals were accessed conventionally by preparing endodontic cavities with high-speed Endo Access burs (Dentsply Sirona, Ballaigues, Switzerland) under copious water-cooling. The presence of two separate mesial root canals was confirmed radiographically using the size 10 K-file (Dentsply Sirona, Ballaigues, Switzerland) inserted to the full working length (WL). The WL of both mesial canals was determined by inserting a size 10 K-file into the root canal until the tip approached the apical foramen and was visible under 10× magnification (OPMI Pico, Carl Zeiss, Oberkochen, Germany). Afterwards, the WL was increased by 2 mm to over-instrument the root canal and simulate apical perforation. All mesial canals were enlarged beyond the apical foramen. The glide path was created using size 15 and 20 K-Flexofiles (Dentsply Sirona, Ballaigues, Switzerland), and the root canal shaping was performed with ProTaper NEXT (Dentsply Sirona, Ballaigues, Switzerland) nickel-titanium rotary instruments at the established WL in the following sequence: X1 (17/0.04), X2 (25/0.06), X3 (30/0.07), X4 (40/0.06) and X5 (50/0.06). Instruments were driven using an X-Smart (Dentsply Sirona, Ballaigues, Switzerland) endodontic motor at the rotation speed of 300 rpm and the torque of 1 Ncm.

After the use of each instrument, root canals were repeatedly irrigated with 5 mL 3% sodium hypochlorite (Ultradent Products Inc., South Jordan, UT, USA), while 5 mL of 18% ethylenediaminetetraacetic acid (Ultradent Products Inc., South Jordan, UT, USA) followed by 5 mL of distilled water was used for the final flush at the end of instrumentation. The irrigants were delivered using 29-G NaviTip needles (Ultradent Products Inc., South Jordan, UT, USA) attached to disposable syringes. Afterwards, the root canals were dried with paper points.

The imitation of surrounding periodontal tissues and the alveolar bone was achieved using prefabricated A-silicone (3M ESPE, Seefeld, Germany) blocks. Specimens were fixed in these blocks up to the cement-enamel junction after the coverage of apices with a polytetrafluoroethylene tape (Tesa SE, Norderstedt, Germany).

### 2.2. Root Canal Obturation

A true randomness generator (www.random.org, accessed on 25 October 2021) was used for random allocation of the samples into four equal experimental groups (10 teeth/20 canals per group), according to the material and technique selected and used for root canal obturation:

- BR/SC group—the root canals were filled with BioRoot RCS sealer and single ProTaper NEXT size X5 gutta-percha point (Dentsply Sirona, Ballaigues, Switzerland). The apical 4 mm of the gutta-percha point was cut with a sterile scalpel to fit the gutta-percha with a tug-back effect at a length 2 mm shorter than the perforated apical foramen. The sealer was mixed according to the manufacturer’s instructions, inserted into the Skini syringe (Ultradent Products Inc., South Jordan, UT, USA) and subsequently delivered into the root canal via attached plastic Capillary Tip cannula (Ultradent Products Inc., South Jordan, UT, USA). The tip was inserted approximately 2 mm shorter than the perforation site, and the plunger of the syringe was gently pressed while withdrawing the plastic cannula until reaching the orifice level. After the injection of BioRoot RCS, the pre-fitted gutta-percha point was coated with a thin amount of the sealer and gently inserted into the root canal 2 mm short of the perforated apex.
- BR/SC-UA group—the root canals were filled with BioRoot RCS sealer and single ProTaper NEXT size X5 gutta-percha point using ultrasonic agitation. The selection and adaptation of the gutta-percha point and the injection of the sealer were accomplished identically to the BR/SC group. After delivering the sealer into the root canal, an Ultrawave ET25 ultrasonic tip (Ultradent Products Inc., South Jordan, UT, USA) attached to an Ultrawave XS ultrasonic device (Ultradent Products Inc., South Jordan, UT, USA) was directly inserted into the root canal and BioRoot RCS sealer 2 mm short of the WL. The ultrasonic tip was activated for 10 s at the medium power using Reflex technology (Ultradent Products Inc.), capable of automatic real-time frequency adjustment of 28–36 kHz. The pre-fitted gutta-percha point was subsequently coated with a small amount of the sealer and slowly inserted into the root canal 2 mm shorter than the apical foramen.
- MF group—the root canals were filled with MTA Flow cement. A total of 0.19 g of powder and 3 drops of liquid were mixed according to the manufacturer’s recommendations to get a thin consistency of the cement. The mixed material was inserted into the clear Skini syringe, and the flowability of the material was checked by extruding the small amount of the cement through the attached 29-G NaviTip needle. The filling material was delivered into the root canal by slowly pressing the plunger of the syringe and withdrawing the tip, which was inserted 2 mm short of the perforated apex.
- MF-UA group—the root canals were filled with MTA Flow cement using ultrasonic agitation. The filling material was prepared and injected into the root canal in the same manner as in the MF group. Afterwards, the Ultrawave ET25 ultrasonic tip was directly inserted into the root canal and MTA Flow cement 2 mm short of the perforation site and activated for 10 s at the 28–36 kHz frequency and the power described previously.

Postoperative radiographs were made immediately after the obturation of the root canals to evaluate the filling quality. The obturation procedure was repeated when a lack of homogeneity or inadequate filling length was observed. New radiographs were taken to confirm the quality of the root canal fillings afterwards. The heat carrier was used to cut the gutta-percha point at the orifice level in the BR/SC and BR/SC-UA groups. The endodontic access cavities of all specimens were sealed with temporary filling material Cavit™-W (3M ESPE, Seefeld, Germany), and the teeth were stored at 37 °C and 100% humidity for 7 days to allow the filling materials to set completely.

All specimens were prepared and obturated by a single operator: an experienced endodontist.

### 2.3. Micro-CT Analysis

Teeth were scanned before and after root canal obturation with a high-resolution micro-CT scanner SkyScan 1272 (Bruker, Kontich, Belgium). The scanning parameters were set at 100 kV source voltage, 100 µA beam current, 9.9 µm isotropic resolution, 0.11 mm copper filter, 1073 ms exposure time, 0.4° rotation step and 360° rotation angle. The obtained images were reconstructed using NRecon v.1.6.9.18 software (Bruker, Kontich, Belgium) under a beam hardening correction of 20% and a ring artefact reduction factor of 6.

The CTAn v.1.14.4.1 software (Bruker, Kontich, Belgium) was used to analyze the quality of root canal fillings in the apical 5 mm. All grayscale images from the selected region of interest were converted to binary images using a global threshold method in a density histogram. The original and segmented scans were thoroughly compared to confirm the segmentation accuracy before further analysis with a custom-processing tool. Images obtained from pre-obturation scans were used for quantification of the root canal volume (C_Vol_), while post-obturation images were used to determine volumes of filling material (F_Vol_) and closed pores (CP_Vol_). The total volume of pores (V_Vol_) and volume of open pores (OP_Vol_) were calculated using the following formulas, respectively:

V_Vol_ = C_Vol_ − F_Vol_,

OP_Vol_ = V_Vol_ − CP_Vol_.

Afterwards, the percentage volume of open (%OP_Vol_) and closed (%CP_Vol_) pores was determined as follows:

%OP_Vol_ = OP_Vol_/C_Vol_ × 100,

%CP_Vol_ = CP_Vol_/C_Vol_ × 100

The evaluation of micro-CT images was performed by a single person who was blinded to data regarding the root canal filling material and technique.

### 2.4. Statistical Analysis

The porosity distribution between experimental groups was compared using a non-parametric Kruskal-Wallis test followed by the Mann-Whitney test due to a non-normal distribution of the data and validated with the Shapiro-Wilk test. All comparisons were performed using SPSS 25.0 software (SPSS Inc., Chicago, USA), with the significance level set at *p* < 0.05.

## 3. Results

None of the techniques used was able to provide a pore-free root canal filling in the apical 5 mm pores; size and shape diversity were observed in all apical plugs, with open pores being the dominant type of porosity. The results of quantitative volumetric analysis of open and closed pores are summarized in Table 1. The micro-CT assessment revealed that volumes of prepared root canals had no considerable volumetric variances before the root obturation procedure (*p* = 0.34), indicating the initial equality among all experimental groups. However, the porosity distribution in root canal fillings was significantly different between all experimental groups evaluated (*p* < 0.05).

**Figure 1:**
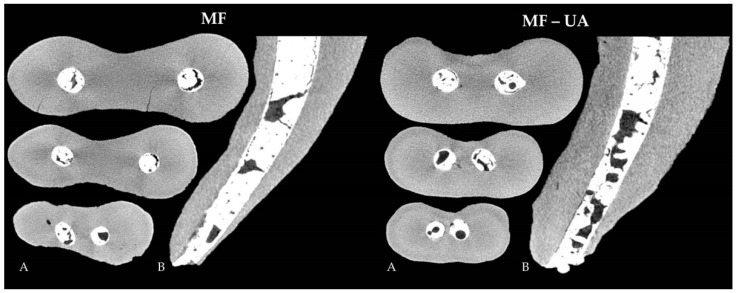
Representative cross-sections of random samples at the level of 5 mm, 3 mm and 1 mm from the apex (**A**) and longitudinal sections (**B**), demonstrating the porosity distribution within the fillings of MF (MTA Flow) and MF-UA (MTA Flow with ultrasonic agitation) groups.

A considerably higher quantity of open and closed pores was observed in both MF groups (with/without ultrasonic agitation), when compared to the fillings of BR/SC and BR/SC-UA (*p* < 0.05). The interaction between the MF and MF-UA groups was detected to be statistically significant (*p* < 0.05), with the highest porosity being in the MF-UA group (Figure 1).

**Figure 2:**
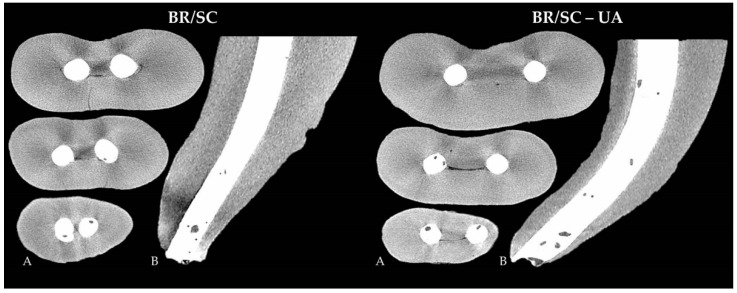
Cross-sectional images at the level of 5 mm, 3 mm and 1 mm from the apex (**A**) and longitudinal images (**B**), representing the quality and homogeneity of BR/SC (BioRoot RCS/single cone) and BR/SC-UA (BioRoot RCS/single cone with ultrasonic agitation) apical plugs.

However, no significant differences were observed between the specimens of the BR/SC and BR/SC-UA groups, where the quantity of open and closed pores within the fillings remained similar (*p* = 0.82 and *p* = 0.57, respectively) regardless of a lower mean porosity determined in the BR/SC group (Figure 2).

## 4. Discussion

Root perforations are one of the most common complications observed in modern endodontology [4]. Regardless of recent advances in the field of endodontic instruments and devices, the mechanical preparation of curved root canals still remains a significant challenge, even for experienced clinicians [30]. It has been reported that the risk of root perforation occurring strongly correlates with the degree of root canal curvature, and the prevalence of apical root perforations is significantly higher in molars as compared to other teeth [2,31]. Therefore, mandibular first molars with a moderate curvature of mesial roots were selected in the present study to maximize its clinical relevance.

The management of root perforation is a time-dependent procedure, where hermetic physical seal is crucial to improve the prognosis and survival of the affected tooth [32]. It has been reported that up to 52–79% of the root canal may remain unprepared, regardless of the instruments or instrumentation technique used [33], and no currently available irrigation protocol is capable of completely disinfecting the entire root canal system [34]. Therefore, the obturation phase of the endodontic treatment has undeniable importance in order to create an unfavorable environment for the microorganisms left inside the root canal system after the preparation and to prevent their penetration into periapical tissues [4,35]. The homogeneity of root canal obturation highly depends on the porosity of the fillings [25], as open pores communicating with dentinal walls may create an excellent pathway for microleakage and eventually decrease the success rate and outcome of endodontic treatment [28,36]. Closed pores are considered to be less clinically relevant, as they represent empty spaces completely surrounded by filling material [37]. Nevertheless, it has been shown that this type of porosity may negatively affect the physical properties of the material, such as hardness and strength [36,38]. Therefore, the quantification of both open and closed pores is necessary to evaluate the quality of root canal fillings properly. Previously, various porosity and leakage measuring approaches, such as dye staining, glucose or radioactive isotope penetration, protein loss, scanning electron microscopy, mercury and capillary flow porometry, were applied to assess the sealing feature of the material used [39]. However, the significant limitations of these methods, e.g., the need to section the samples and hence the creation of artifacts, led to micro-CT being the technique of choice for accurate 3D evaluation of root canal fillings [20]. Therefore, micro-CT analysis was used in the present study to quantify and qualify the pores within the apical plugs. The isotropic resolution was set at 9.9 µm, as it has been shown that a voxel size of 11.2 μm or less is a reliable cutoff value to assess the filling porosity [36,40], even though there is always a risk of tiny pores left undetected due to a high radiopacity of the material used [18].

Techniques and materials applied for root perforation repair have not been standardized. However, MTA is generally assumed to be a benchmark for sealing various types of root perforations [32]. MF is one of the newest MTA-based repair materials, which surpasses traditional MTA in terms of clinical applicability due to its superior handling and delivery characteristics as well as faster setting time and thus increased washout resistance [10,19]. Moreover, MF retains all desirable biological properties of the original MTA, such as biocompatibility and bioactivity, which are the crucial requirements for perforation repair material exposed to periodontal tissues [41]. The biocompatibility and bioactivity are attributed mainly to the continuous calcium ion release and the formation of calcium phosphate apatite crystals, which induce the regeneration and remineralization of adjacent hard tissues while also reducing the porosity of filling material [28,41]. Nevertheless, a previous study has shown that, despite all the improvements and advantageous characteristics, MF results in highly porous apical plugs [19]. These results are in accordance with the present study, in which both MF groups (with/without ultrasonic agitation) exhibited a high porosity. The incidence of pores within MF fillings can be attributed to the increased water-to-cement ratio used during the mixing procedure to achieve a highly flowable consistency of the cement. It has been reported that excess water in the mixture eventually dries off and leaves pores that are not filled by hydration products [42]. Additionally, bismuth oxide, which is added to the MF composition as a radiopacifier, can negatively affect the sealing features by interfering with the hydration reaction and leaving more unreacted water within the filling [43]. Instead of bismuth oxide, some HCSC formulations, e.g., BioRoot RCS, contain zirconium oxide, which appears to have no impact on the material porosity [44]. These findings may correlate with the results of the present study, where significantly more homogeneous apical plugs were observed in both BR/SC groups than the MF groups.

Sealing apical root perforations with BioRoot RCS, together with a modified SC obturation technique, was proposed mainly due to the simplicity and effectiveness previously reported in in vitro and in vivo studies [16,17,19]. The concept of the SC obturation technique refers to the desirable physico-chemical properties of BioRoot RCS [14,15], which was designed as a biological filler [45], and to the tapered gutta-percha cone, acting as a piston on the flowable sealer [46]. As reported previously, the insertion of the tapered gutta-percha cone creates hydraulic pressure, which improves the material distribution throughout the root canal [47]. Therefore, the gutta-percha cone may be considered as the main factor leading to the significant differences between the BR/SC and MF groups. No porosity associated with gutta-percha cones was observed in the present study through micro-CT analysis. Therefore, the superior overall homogeneity of BR/SC apical plugs can be attributed to the use of solid gutta-percha cones.

Attempts to minimize the occurrence of pores within BR/SC and MF fillings by using ultrasonic agitation were made in our previous study, which demonstrated that neither of these techniques was able to produce pore-free apical plugs [19]. The effect of ultrasonic application mainly refers to the acoustic energy transmission and the formation of cavitation bubbles, which eventually implode, increasing the temperature and the pressure inside the root canal [21]. According to previous investigations, which have reported significantly better results in terms of porosity after the use of indirect ultrasonication, the increased pressure may remove the entrapped air, disperse agglomerated particles, reduce their surface friction and provide a more efficient incorporation of filler particles into the organic matrix, with no changes in particle size or material composition [23,25,48]. Additionally, the pressure generated during ultrasonic agitation may lead to superior interfacial adaptation between the filling material and the root canal wall, with better tubular penetration as well [21,22]. However, these advantageous effects of ultrasonic application did not provide more homogeneous apical plugs in the present study; the increased percentages of open and closed pores were observed in both BR/SC-UA and MF-UA groups. Therefore, the null hypothesis was rejected.

The lower overall homogeneity of ultrasonicated apical plugs could be attributed to the direct ultrasonic agitation, resulting in excessive vibratory forces. It has been reported that excessive ultrasonic energy potentially can lead to air incorporation into the filling material and thus contribute to the higher porosity [26,49]. However, the use of direct ultrasonic agitation should not be directly associated with less homogenous root canal fillings, since it has been reported that indirect ultrasonication may also increase the porosity [20]. Instead of ultrasonication type, more attention should be paid to the agitation time, which is potentially directly related to both the rearrangement of cement particles and the heat generation [20,50]. The ultrasonic agitation of 10 s was selected in the present study in accordance with Sisli et al. [8], who agitated 5 mm apical plugs for 10 s and afterwards reported a lower incidence of pores. It has been reported that a short agitation time may create a shock-like effect, and the duration of 5 to 10 s is necessary to rearrange the cement particles and decrease the porosity [20]. On the other hand, the prolonged agitation time may be responsible for the increase in temperature, ultimately leading to water loss from HCSC [22,49]. Even though the number of published studies evaluating the temperature changes in filling materials is still limited, there are few reports in the literature indicating that ultrasonic agitation is capable of raising the temperature inside the root canal by 2 °C [7], which can be sufficient to increase the water desorption occurring at temperatures as low as 20 °C [51]. The water loss may alter the rheological properties of the material and increase the porosity [7,51], which is considered the result of spaces between unhydrated cement particles [42]. Nevertheless, it can be speculated that indirect ultrasonic application is not prone to these adverse effects of temperature changes, as ultrasonic energy is transmitted to the material through the gutta-percha cone, plugger or another instrument. This would explain the contradictory findings in terms of porosity obtained between the present study and previous investigations [8,24], which also performed ultrasonic agitation for 10 s. However, it is difficult to directly compare the present study results with the available literature, as they differ in too many aspects, including the type and properties of filling material, the application technique, ultrasonication type and duration, assessment method, etc.

The present study suggests that all apical plugs, regardless of the obturation technique used, may potentially lead to microleakage, as none of the fillings was pore-free, and the percentages of open pores surpassed the closed porosity in all experimental groups. Nevertheless, MF apical plugs (with/without ultrasonic agitation) demonstrated significantly higher percentages of open and closed pores as compared to the BR/SC obturation technique. Therefore, reinforcing the findings of Benavides-García et al. [52], it can be concluded that MF prepared in a thin consistency should not be the material of choice for apical root perforation repair. Even though there is still no clear evidence what porosity level is critical, the significantly higher amount of pores observed in both MF groups may theoretically contribute to a worse outcome of endodontic treatment [27,28]. On the other hand, it has been shown that HCSC reduces their porosity with time in the presence of tissue fluids [42]. Therefore, the results of the present study should be evaluated with caution, as it is impossible to fully reproduce the clinical conditions using in vitro models. Further studies are needed to determine the clinical efficacy of BR/SC and MF obturation techniques in apically perforated and moderately curved roots and to confirm the adverse impact of direct ultrasonic agitation on the quality and homogeneity of root canal fillings.

## 5. Conclusions

Within the limitations of this in vitro study, it can be concluded that none of the obturation techniques was able to provide pore-free root canal fillings in the apical 5 mm. Significantly higher porosity was observed in the MF and MF-UA groups as compared to the BR/SC and BR/SC-UA groups. The direct ultrasonic agitation had no considerable impact on the porosity distribution in BR/SC fillings, while MF fillings demonstrated significantly higher overall porosity after ultrasonic agitation.

## Figures and Tables

**Table 1:** Mean values (%) and standard deviations (SD) of open and closed pores in the respective groups.

Group	*N*	Open Pores	Closed Pores
BR/SC	20	3.374 ± 2.751 ^A^	0.061 ± 0.080 ^A^
BR/SC-UA	20	3.390 ± 3.428 ^A^	0.066 ± 0.070 ^A^
MF	20	18.832 ± 3.334 ^B^	0.292 ± 0.226 ^B^
MF-UA	20	29.075 ± 9.440 ^C^	0.923 ± 0.684 ^C^

Different superscript letters in the same column indicate significant differences between groups (pairwise Mann-Whitney test; *p* < 0.05).

## Data Availability

Data is contained within the article.

## References

[1] Bueno M.R., Estrela C., De Figueiredo J.A., Azevedo B.C. (2011). Map-reading strategy to diagnose root perforations near metallic intracanal posts by using cone beam computed tomography. J. Endod..

[2] Sarao S.K., Berlin-Broner Y., Levin L. (2021). Occurrence and risk factors of dental root perforations: A systematic review. Int. Dent. J..

[3] Siew K., Lee A.H., Cheung G.S. (2015). Treatment outcome of repaired root perforation: A systematic review and meta-analysis. J. Endod..

[4] Gorni F.G., Andreano A., Ambrogi F., Brambilla E., Gagliani M. (2016). Patient and clinical characteristics associated with primary healing of iatrogenic perforations after root canal treatment: Results of a long-term Italian study. J. Endod..

[5] Estrela C., Decurcio D.A., Rossi-Fedele G., Silva J.A., Guedes O.A., Borges Á.H. (2018). Root perforations: A review of diagnosis, prognosis and materials. Braz. Oral. Res..

[6] Tsesis I., Fuss Z. (2006). Diagnosis and treatment of accidental root perforations. Endod. Top..

[7] Lopes F.C., Zangirolami C., Mazzi-Chaves J.F., Silva-Sousa A.C., Crozeta B.M., Silva-Sousa Y.T.C., Sousa-Neto M.D. (2019). Effect of sonic and ultrasonic activation on physicochemical properties of root canal sealers. J. Appl. Oral. Sci..

[8] Sisli S.N., Ozbas H. (2017). Comparative micro-computed tomographic evaluation of the sealing quality of ProRoot MTA and MTA Angelus apical plugs placed with various techniques. J. Endod..

[9] Mondelli J.A.S., Hoshino R.A., Weckwerth P.H., Cerri P.S., Leonardo R.T., Guerreiro-Tanomaru J.M., Tanomaru-Filho M., da Silva G.F. (2019). Biocompatibility of mineral trioxide aggregate flow and biodentine. Int. Endod. J..

[10] Guimarães B.M., Vivan R.R., Piazza B., Alcalde M.P., Bramante C.M., Duarte M.A.H. (2017). Chemical-physical properties and apatite-forming ability of mineral trioxide aggregate flow. J. Endod..

[11] Ultradent Ultradent Products, Inc. Proudly Introduces MTA Flow™ Repair Cement. https://www.ultradent.com/company/newsroom/article/ultradent-products-inc-proudly-introduces-mta-flow-repair-cement.

[12] Liu M., He L., Wang H., Su W., Li H. (2021). Comparison of in vitro biocompatibility and antibacterial activity of two calcium silicate-based materials. J. Mater. Sci. Mater. Med..

[13] Drukteinis S., Drukteinis S., Camilleri J. (2021). Bioceramic Materials for Management of Endodontic Complications. Bioceramic Materials in Clinical Endodontics.

[14] Siboni F., Taddei P., Zamparini F., Prati C., Gandolfi M.G. (2017). Properties of BioRoot RCS, a tricalcium silicate endodontic sealer modified with povidone and polycarboxylate. Int. Endod. J..

[15] Sfeir G., Zogheib C., Patel S., Giraud T., Nagendrababu V., Bukiet F. (2021). Calcium silicate-based root canal sealers: A narrative review and clinical perspectives. Materials.

[16] Bardini G., Casula L., Ambu E., Musu D., Mercadè M., Cotti E. (2021). A 12-month follow-up of primary and secondary root canal treatment in teeth obturated with a hydraulic sealer. Clin. Oral Investig..

[17] Zavattini A., Knight A., Foschi F., Mannocci F. (2020). Outcome of root canal treatments using a new calcium silicate root canal sealer: A non-randomized clinical trial. J. Clin. Med..

[18] Drukteinis S., Bilvinaite G., Tusas P., Shemesh H., Peciuliene V. (2021). Microcomputed tomographic assessment of the single cone root canal fillings performed by undergraduate student, postgraduate student and specialist endodontist. J. Clin. Med..

[19] Drukteinis S., Peciuliene V., Shemesh H., Tusas P., Bendinskaite R. (2019). Porosity distribution in apically perforated curved root canals filled with two different calcium silicate based materials and techniques: A micro-computed tomography study. Materials.

[20] El-Ma’aita A.M., Qualtrough A.J., Watts D.C. (2012). A micro-computed tomography evaluation of mineral trioxide aggregate root canal fillings. J. Endod..

[21] da Silva Machado A.P., Câncio Couto de Souza A.C., Lima Gonçalves T., Franco Marques A.A., da Fonseca Roberti Garcia L., Antunes Bortoluzzi E., Acris de Carvalho F.M. (2021). Does the ultrasonic activation of sealer hinder the root canal retreatment?. Clin. Oral Investig..

[22] Aguiar B.A., Frota L.M.A., Taguatinga D.T., Vivan R.R., Camilleri J., Duarte M.A.H., de Vasconcelos B.C. (2019). Influence of ultrasonic agitation on bond strength, marginal adaptation, and tooth discoloration provided by three coronary barrier endodontic materials. Clin. Oral Investig..

[23] Wiesse P.E.B., Silva-Sousa Y.T., Pereira R.D., Estrela C., Domingues L.M., Pécora J.D., Sousa-Neto M.D. (2018). Effect of ultrasonic and sonic activation of root canal sealers on the push-out bond strength and interfacial adaptation to root canal dentine. Int. Endod. J..

[24] Dinçer A.N., Güneşer M.B., Sisli S.N. (2020). Micro-CT analysis of the marginal adaptation and porosity associated with ultrasonic activation of coronally placed tricalcium silicate-based cements. Aust. Endod. J..

[25] Kim J.A., Hwang Y.C., Rosa V., Yu M.K., Lee K.W., Min K.S. (2018). Root Canal Filling Quality of a Premixed Calcium Silicate Endodontic Sealer Applied Using Gutta-percha Cone-mediated Ultrasonic Activation. J. Endod..

[26] Kim S.Y., Jang Y.E., Kim B.S., Pang E.K., Shim K., Jin H.R., Son M.K., Kim Y. (2021). Effects of ultrasonic activation on root canal filling quality of single-cone obturation with calcium silicate-based sealer. Materials.

[27] An H.J., Yoon H., Jung H.I., Shin D.H., Song M. (2021). Comparison of obturation quality after MTA orthograde filling with various obturation techniques. J. Clin. Med..

[28] Milanovic I., Milovanovic P., Antonijevic D., Dzeletovic B., Djuric M., Miletic V. (2020). Immediate and long-term porosity of calcium silicate-based sealers. J. Endod..

[29] Schneider S.W. (1971). A comparison of canal preparations in straight and curved root canals. Oral Surg. Oral Med. Oral Pathol..

[30] Ansari I., Maria R. (2012). Managing curved canals. Contemp Clin. Dent..

[31] Schafer E., Dammaschke T. (2006). Development and sequelae of canal transportation. Endod. Top..

[32] Abdelmotelb M.A., Gomaa Y.F., Khattab N.M.A., Elheeny A.A.H. (2021). Premixed bioceramics versus mineral trioxide aggregate in furcal perforation repair of primary molars: In vitro and in vivo study. Clin. Oral Investig..

[33] Lopes R.M.V., Marins F.C., Belladonna F.G., Souza E.M., De-Deus G., Lopes R.T., Silva E.J.N.L. (2018). Untouched canal areas and debris accumulation after root canal preparation with rotary and adaptive systems. Aust. Endod. J..

[34] Nagendrababu V., Jayaraman J., Suresh A., Kalyanasundaram S., Neelakantan P. (2018). Effectiveness of ultrasonically activated irrigation on root canal disinfection: A systematic review of in vitro studies. Clin. Oral Investig..

[35] Selem L.C., Li G.H., Niu L.N., Bergeron B.E., Bortoluzzi E.A., Chen J.H., Pashley D.H., Tay F.R. (2014). Quality of obturation achieved by a non-gutta-percha-based root filling system in single-rooted canals. J. Endod..

[36] Torres F.F.E., Guerreiro-Tanomaru J.M., Bosso-Martelo R., Espir C.G., Camilleri J., Tanomaru-Filho M. (2019). Solubility, porosity, dimensional and volumetric change of endodontic sealers. Braz. Dent. J..

[37] Dioguardi M., Quarta C., Sovereto D., Troiano G., Zhurakivska K., Bizzoca M.E., Lo Muzio L., Lo Russo L. (2021). Calcium silicate cements vs. epoxy resin based cements: Narrative review. Oral.

[38] Antonijevic D., Zelic K., Djuric M. Novel calcium silicate based dental material with the addition of biologically active soy compound. Proceedings of the 2015 IEEE 15th International Conference on Bioinformatics and Bioengineering (BIBE).

[39] Guerrero F., Berástegui E. (2018). Porosity analysis of MTA and Biodentine cements for use in endodontics by using micro-computed tomography. J. Clin. Exp. Dent..

[40] Orhan K., Jacobs R., Celikten B., Huang Y., de Faria Vasconcelos K., Nicolielo L.F.P., Buyuksungur A., Van Dessel J. (2018). Evaluation of threshold values for root canal filling voids in micro-CT and nano-CT images. Scanning.

[41] Bueno C.R.E., Vasques A.M.V., Cury M.T.S., Sivieri-Araújo G., Jacinto R.C., Gomes-Filho J.E., Cintra L.T.A., Dezan-Junior E. (2019). Biocompatibility and biomineralization assessment of mineral trioxide aggregate flow. Clin. Oral Investig..

[42] Camilleri J., Grech L., Galea K., Keir D., Fenech M., Formosa L., Damidot D., Mallia B. (2014). Porosity and root dentine to material interface assessment of calcium silicate-based root-end filling materials. Clin. Oral Investig..

[43] Coomaraswamy K.S., Lumley P.J., Hofmann M.P. (2007). Effect of bismuth oxide radioopacifier content on the material properties of an endodontic Portland cement-based (MTA-like) system. J. Endod..

[44] Li X., Yoshihara K., De Munck J., Cokic S., Pongprueksa P., Putzeys E., Pedano M., Chen Z., Van Landuyt K., Van Meerbeek B. (2017). Modified tricalcium silicate cement formulations with added zirconium oxide. Clin. Oral Investig..

[45] Camilleri J. (2019). BioRoot RCS. Endo Sealer or Biological Filler?. https://www.septodont.com.ru/sites/ru/files/2019-07/Septodont_BioRoot_Endo%20sealer%20or%20biological%20filler_JC.pdf.

[46] Camilleri J. (2017). Will bioceramics be the future root canal filling materials?. Curr. Oral Health Rep..

[47] Kalantar Motamedi M.R., Mortaheb A., Zare Jahromi M., Gilbert B.E. (2021). Micro-CT evaluation of four root canal obturation techniques. Scanning.

[48] Acris De Carvalho F.M., Silva-Sousa Y.T.C., Saraiva Miranda C.E., Miller Calderon P.H., Barbosa A.F.S., Domingues De Macedo L.M., Abi Rached-Junior F.J. (2021). Influence of ultrasonic activation on the physicochemical properties of calcium silicate-based cements. Int. J. Dent..

[49] Yeung P., Liewehr F.R., Moon P.C. (2006). A quantitative comparison of the fill density of MTA produced by two placement techniques. J. Endod..

[50] Pérez-Alfayate R., Algar-Pinilla J., Mercade M., Foschi F. (2021). Sonic activation improves bioceramic sealer’s penetration into the tubular dentin of curved root canals: A confocal laser scanning microscopy investigation. Appl. Sci..

[51] Atmeh A.R., AlShwaimi E. (2017). The effect of heating time and temperature on epoxy resin and calcium silicate-based endodontic sealers. J. Endod..

[52] Benavides-García M., Hernández-Meza E., Reyes-Carmona J. (2021). Ex vivo analysis of MTA FLOW^®^ biomineralization and push-out strength: A pilot study. Int. J. Dent. Sci..

